# Evaluation of Epidemic Intelligence Systems Integrated in the Early Alerting and Reporting Project for the Detection of A/H5N1 Influenza Events

**DOI:** 10.1371/journal.pone.0057252

**Published:** 2013-03-05

**Authors:** Philippe Barboza, Laetitia Vaillant, Abla Mawudeku, Noele P. Nelson, David M. Hartley, Lawrence C. Madoff, Jens P. Linge, Nigel Collier, John S. Brownstein, Roman Yangarber, Pascal Astagneau, on behalf of the Early Alerting, Reporting Project of the Global Health Security Initiative

**Affiliations:** 1 International Department, French Institute for Public Health Surveillance (Institut de Veille Sanitaire), Saint Maurice, France; 2 Situational Awareness Section, Public Health Agency of Canada, Ottawa, Canada; 3 Department of Pediatrics, Georgetown University Medical Center, Washington, D.C., United States of America; 4 Department of Microbiology and Immunology, Georgetown University Medical Center, Washington, D.C, United States of America; 5 ProMED-mail, International Society for Infectious Diseases, Boston, Massachusetts, United States of America; 6 Joint Research Centre of the European Commission, Ispra, Italy; 7 National Institute of Informatics, Tokyo, Japan; 8 Boston Children's Hospital, Harvard Medical School, Boston, Massachusetts, United States of America; 9 Department of Computer Science, University of Helsinki, Helsinki, Finland; 10 Department of Public Health, Pierre et Marie Curie University School of Medicine, Paris, France; University of Hong Kong, Hong Kong

## Abstract

The objective of Web-based expert epidemic intelligence systems is to detect health threats. The Global Health Security Initiative (GHSI) Early Alerting and Reporting (EAR) project was launched to assess the feasibility and opportunity for pooling epidemic intelligence data from seven expert systems. EAR participants completed a qualitative survey to document epidemic intelligence strategies and to assess perceptions regarding the systems performance. Timeliness and sensitivity were rated highly illustrating the value of the systems for epidemic intelligence. Weaknesses identified included representativeness, completeness and flexibility. These findings were corroborated by the quantitative analysis performed on signals potentially related to influenza A/H5N1 events occurring in March 2010. For the six systems for which this information was available, the detection rate ranged from 31% to 38%, and increased to 72% when considering the virtual combined system. The effective positive predictive values ranged from 3% to 24% and F1-scores ranged from 6% to 27%. System sensitivity ranged from 38% to 72%. An average difference of 23% was observed between the sensitivities calculated for human cases and epizootics, underlining the difficulties in developing an efficient algorithm for a single pathology. However, the sensitivity increased to 93% when the virtual combined system was considered, clearly illustrating complementarities between individual systems. The average delay between the detection of A/H5N1 events by the systems and their official reporting by WHO or OIE was 10.2 days (95% CI: 6.7–13.8). This work illustrates the diversity in implemented epidemic intelligence activities, differences in system's designs, and the potential added values and opportunities for synergy between systems, between users and between systems and users.

## Introduction

Epidemic intelligence provides a new approach to address the challenges of disease globalization [1]–[3]. It provides an approach that is complementary to countries' national surveillance strategies. Moreover epidemic intelligence was included by the World Health Organization (WHO) in the health threat detection mechanisms integrated into the International Health Regulations [4], [5]. While epidemiological indicator-based surveillance relies on regular reporting of a number of well-defined indicators provided mainly by health care facilities, epidemic intelligence focuses on event detection, prior to official health care reporting, laboratory confirmation and eventual official notification. Epidemic intelligence consists of the *ad hoc* detection and interpretation of unstructured information available in the Internet. This information is very diverse in nature and is generated by multiple types of sources, both official and informal. The information may include unverified rumors from the media or more reliable information from official sources or traditional epidemiological surveillance systems. These raw signals usually contain very little information (e.g. medical or scientific) on which analysis can be performed, and they are often embedded in noise. Epidemic intelligence is a complex, time and resource-intensive process that includes a formalized protocol for event selection, verification of the genuineness of reported events, searches of complementary reliable information, analysis and communication.

Epidemic intelligence is still a relatively new discipline that emerged in the 1990s triggered by the development of the Internet. ProMED-mail [6] was the first Internet-based reporting system to use both formal and informal sources. It was followed by several expert systems developed to detect relevant information from the Internet [7]–[10]. In parallel, national and international institutions have developed epidemic intelligence capacities to fulfill their own needs [8], [11]–[14]. A number of studies [15]–[18] have been carried out to assess expert systems' abilities to detect and correctly classify health threats using informal open sources or to present innovative functionalities. These papers rarely address users' viewpoints (i.e., the detection of relevant information by public health institutions). A thorough evaluation of epidemic intelligence information faces major challenges, including the lack of an adequate gold standard and standardized indicators and, but also the type of information collected, which is often not designed for health surveillance purposes.

The development of expert systems and epidemic intelligence took place independently, resulting in both the development of specific expertise among expert systems and institutions and, varying degrees of duplication. This paper aims to present a methodology and results that can be utilized to assess the complementarity of expert systems' capability and epidemic intelligence frameworks.

## Methods

### The EAR Project

The Global Health Security Initiative (GHSI) is an informal, international partnership among like-minded countries aiming to strengthen global health preparedness and response to chemical, biological, radio-nuclear (CBRN) terrorism and pandemic influenza threats. GHSI was launched in November 2001 by Canada, the European Union (EU), France, Germany, Italy, Japan, Mexico, the United Kingdom and the United States. The WHO serves as an expert advisor to the GHSI [19]. In 2009, an international project called Early Alerting and Reporting (EAR) was established, bringing together end-users (i.e., public health institutions in charge of epidemic intelligence), systems providers, and stakeholders (see Tables 1 and 2). Its objective for 2009–2010 was to assess the feasibility of developing a single web-based platform that would enable partners to access health threats identified from open source web-based public health intelligence systems, as well as to combine risk assessment processes.

**Table 1:** Early Alerting and Reporting (EAR), participating systems.

	System name	System owner/developer	Country	Moderation type	n users 2010*	references
**Expert systems**	Argus	Georgetown University	USA	Human moderation	5	[29]–[31]
	BioCaster	National Institute of Informatics	Japan	Fully automated	4	[8], [16], [32]
	GPHIN	Public Health Agency of Canada	Canada	Human moderation	6	[1], [9], [33]
	HealthMap	Harvard University	USA	Partially moderated	5	[7], [34], [35]
	MedISys	Joint Research Centre	EU	Fully automated	5	[10], [36], [37]
	ProMED-mail	International Society of Infectious Diseases	USA	Human moderation	9	[6], [17], [23]
	Puls	University of Helsinki	Finland	Fully automated	4	[18], [38], [39]

**Table 2:** Early Alerting and Reporting (EAR) public health institutions and stakeholders.

	Institution name	Country
Public Health Institutions	Centers for Disease Control and Prevention (CDC)	United States (USA)
	European Centre for Disease Prevention and Control (ECDC)	European Union (EU)
	Health Protection Agency (HPA)	United Kingdom
	Institut de Veille Sanitaire (InVS)	France
	Istituto Superiore di Sanità (ISS)	Italy
	National Institute of Infectious Diseases (NIID)	Japan
	Public Health Agency of Canada (PHAC)	Canada
	Robert Koch Institute (RKI)	Germany
Stakeholders	Ministries of Health	Canada
		France
		Germany
		Italy
		Japan
		Mexico
		United Kingdom
		United States
	Directorate General for Health and Consumers of the European Commission (DG-SANCO)	
	European Food Safety Authority (EFSA)	
	World Health Organization (WHO) as observer	

### Evaluation

The study included a qualitative (questionnaire-based) and a quantitative assessment. The qualitative assessment's goal was to provide information essential for determining the best strategy for the quantitative part of the study.

### Qualitative analysis

A questionnaire was constructed to assess both the type of epidemic intelligence performed by participating public health institutions and their perception of the seven integrated expert systems. The questionnaire, sent to ten EAR points of contact, was self-administrated during the first quarter of 2010. In order to measure the perceived performances of each system that they at least occasionally utilize, users were asked to rate each system through a simple choice (Yes/No). The following pre-defined characteristics were measured: representativeness (of information e.g., geographic coverage, type of diseases, etc.), completeness (or “exhaustivity” of information collected for the detected events), timeliness (of reporting), sensitivity (of the system), usefulness (of provided information), simplicity of use and flexibility of the systems (adaptation to users needs'). Results were represented in two-dimensional spider-charts of the proportion of users that selected the corresponding attribute to describe the system (denominator being the number of systems' users). Participants were also requested to describe epidemic intelligence activities implemented in their institution in terms of priority domains of interest (e.g., specific epidemic diseases, plant diseases, CBRN agents, and natural disasters), the selection criteria, and frequency of these activities.

### Gold standard

A review was performed to identify a global health threat covered by all participating expert systems, which was frequently occurring and for which a “gold standard” exists. Highly pathogenic avian influenza A/H5N1 (A/H5N1) was selected as the most appropriate. Human and veterinary cases are subjected to mandatory notification through WHO and the World Organisation for Animal Health (OIE). These two international organizations constitute the most reliable and recognized source of information regarding A/H5N1 biologically confirmed human cases and epizootics. Human cases and epizootics (both single cases of animal disease and larger outbreaks) that occurred in March 2010 (date of first symptoms, date of the start of the outbreak) or reported by WHO/OIE in March 2010 were considered as the gold standard.

### Quantitative analysis: Database and indicator analyses

#### Raw data

Despite intrinsic differences, expert systems operate in similar ways. They search the Internet to detect information potentially relevant for epidemic intelligence purposes, i.e., raw signals in unprocessed news articles, messages in forums, official press releases, extracts from public official websites, etc. These signals are then stored on dedicated web-based platforms (specific to each system) accessible to end-users for their assessment and verification.

#### Databases

Two different databases were constituted: The first one (“prospective”) aimed at assessing the event detection process under close to real life conditions (i.e., detecting pertinent signals potentially relevant for the study among a large volume of raw signals). The second (“retrospective”) database aimed at assessing systems' theoretical performances.

#### “Prospective” database: Detection rate, Effective Positive Predictive Value (EPPV), F1-score

All raw signals detected between March 1 and March 31, 2010 that potentially referred to an A/H5N1 event (human cases and epizootics) were considered for the analysis. Raw signals were automatically collected (through prospective specifically designed queries), provided directly by the systems (i.e. datasets), or collected manually (through retrospective *ad hoc* queries). From these data, each signal captured by the systems was reviewed and classified as: detected or inadequately detected. A detected (DET) event was defined as the first report mentioning a human case or an epizootic detected by a system in March 2010 and before the reporting of this event by WHO/OIE on their respective websites. An inadequately detected (XDET)) was defined as a signal initially tagged A/H5N1, but after verification was found to be not related to the occurrence of confirmed A/H5N1 cases, or an event previously detected by the same system (i.e. duplicate), or an A/H5N1 case report detected by a system after or on the same day as the reporting of this event by WHO/OIE. A not-detected (NDET) event was defined as an event reported by WHO/OIE but not detected by the system in March 2010. True negative events could not be considered because it is not possible to determine the total number of reports issued on the Internet nor those events discovered but not published by systems. The detection rate (DR) was defined as the ability of a system to detect confirmed A/H5N1 cases before their reporting by WHO/OIE (DR = DET/(DET+NDET)). Effective Predictive Positive Value (EPPV) was defined as the probability for the system to timely detect confirmed A/H5N1 cases among all reports (EPPV = DET/(DET+XDET)). The F1-score is the harmonic mean of DR and EPPV, weighted equally [16] F1 = 2*(EPPV*DR)/(EPPV+DR).

#### “Retrospective” database: Sensitivity and timeliness

For each event included in the gold standard (i.e., reported by WHO or OIE), a specific manual retrospective search was performed on all systems to identify the first report related to this event. No restriction was set on the time period in order to capture both early and late event detection. A true positive (TP) event was defined as the first report mentioning a human case or an epizootic detected by a system before the reporting of the event by WHO/OIE. A false negative (FN) event was defined as an event not detected by the system. Sensitivity (Se = TP/(TP+FN)) was defined as the retrospective ability of a system to detect an event included in the gold standard. Timeliness was defined as the delay between official reporting and the detection by a system (date of report on WHO/OIE websites minus date of first detection by the system, in days). Common variables were used for the analyses: mean, median, rates. Box plot graphs were made to display timeliness, statistical measures and the ANOVA test was used to compare mean values. All statistics were computed using Stata 11.0 for Windows.

#### Type of events

When used with the terms DR, EPPV, timeliness and Se, “overall” refer to animal and human cases.

#### Virtual combined system

In order to assess the complementarity and added value of combining the systems' information, a virtual system named “combined system” was constructed by pooling signals detected by all systems.

## Results

### Qualitative analysis

Ten users from seven countries and EU public health institutions (Centers for Disease Control and Prevention (CDC), European Commission (EC), European Centre for Disease Prevention and Control (ECDC), Health Protection Agency (HPA), Institut de Veille Sanitaire (InVS), Istituto Superiore di Sanità (ISS), National Institute of Infectious Diseases (NIID), Public Health Agency of Canada (PHAC) and Robert Koch Institute (RKI)) participated in the survey. Respondents were either the head of a unit or an epidemiologist in charge of epidemic intelligence related activities within their institution.

#### Epidemic intelligence focus

According to participants, epidemic intelligence processes varied widely. However, infectious disease was the main focus for most of the experts involved in this survey. All users systematically considered epidemic-prone diseases in general, though, for some institutions the focus was set on specific diseases. Only three countries monitored systematically generic zoonoses in their routine activities. All events involving potential bio-terrorism pathogens were systematically monitored by three countries, while no country included systematically radiological/nuclear and chemical threats in their routine activities. Although, CBRN threats are of interest to all countries, the bio-terrorism aspect (i.e., intentional release) was not considered as pertinent from the detection perspective. Plant diseases were included in the threat detection criteria of one institution. Natural disasters were monitored according to specific criteria (e.g., geographical, type and size of disaster).

#### Variations in system usage

Not all users had access or used routinely all of the expert systems included in the study. Of the seven systems four are freely accessible (BioCaster, HealthMap, MedISys and ProMED-mail) and three have restricted access (Argus, GPHIN and Puls). Nine of the ten respondents utilized regularly at least one of the seven included systems; the remaining respondent used other expert systems not included in this survey. Users routinely accessed from four to seven different systems and their utilization varied greatly. ProMED-mail was used routinely by all respondents while utilization of the other six systems ranged from 60% to 80%. When routinely used, Argus, GPHIN and ProMED-mail were accessed on a daily basis. GPHIN and ProMED-mail were predominantly used for early prospective alert detection (60%), while others were used mostly as a complementary source of information (e.g. to further document already detected events). Finally, 60% of users also utilized other epidemic intelligence systems that were not integrated into the survey, e.g., RSOE-EDIS (Radio Distress-Signaling and Infocommunications, Emergency and Disaster Information Service) or EpiSPIDER [20].

#### Systems' users perception

**Figure 1:**
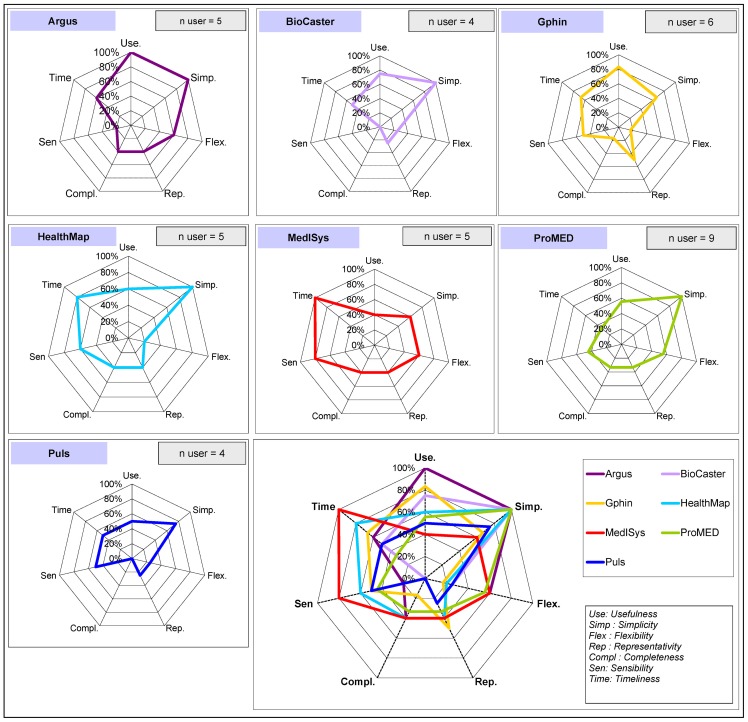
Users' perception regarding systems performances.

The perceptions of users regarding the system attributes (completeness, flexibility, representativeness, sensitivity, simplicity, timeliness and usefulness) are represented Figure 1. Timeliness scores ranged from 33% to 100% and usefulness scores ranged from 40% to 100%. Simplicity was the highest scored attribute with scores ranging from 60% to 100%. Sensitivity ranged from 0% (i.e., no user qualified the system as sensitive) to 80%. The spider charts also highlighted the global weaknesses perceived by users with lower scores for three attributes: flexibility (17% to 60%), representativeness (25 to 50%) and completeness (0 to 40%). Individual spider graphs tend to have relatively similar surfaces, except for the less utilized systems (<5 users).

### Quantitative analysis: A/H5N1 data

#### Detection rate, positive predictive value & F1-score (Table 3)

A total of 1,154 signals potentially relating to A/H5N1 events were collected. For the same period, 29 A/H5N1 events were reported by WHO (14 events) or OIE (15 events) and were included in the gold standard. In regard to large differences in terms of intrinsic systems' characteristics, interface designs, database storage or extraction capacities it has not been possible to implement a homogenous data collection procedure across the seven systems. For one system (GPHIN), the system design did not allow the extraction or collection of data in a format compatible with this analysis and as such 366 signals were excluded from the analysis. As of July 30, 2010, six datasets were collected from the six other systems for a total of 788 signals. Three of these datasets were collected prospectively and three were collected retrospectively.

**Table 3:** Detection rate, positive predictive value and F1 score for A/H5N1 human cases and epizootic detected by systems from 1st to 31^st^ March 2010.

	Systems	Argus	BioCaster	HealthMap	MedISys	ProMED	Puls	Combined system (a)
	Collection process	Auto	Auto	Prov	Prov	Auto	Prov	-
	n signals	103	95	126	347	37	80	788
A/H5N1 human cases (H)	Detected	5	8	6	5	4	5	13
	Not detected	9	6	8	9	10	9	1
	Inadequately detected (b)	14	20	45	52	14	34	179
	Detection rate	36%	57%	43%	36%	29%	36%	93%
	EPPV	26%	29%	12%	9%	22%	13%	7%
	F1 score	30%	38%	18%	14%	25%	19%	13%
A/H5N1 epizootics (V)	Detected	4	3	5	6	5	6	8
	Not detected	11	12	10	9	10	9	7
	Inadequately detected(d)	66	25	39	227	8	19	384
	Detection rate	27%	20%	33%	40%	33%	40%	53%
	EPPV	6%	11%	11%	3%	38%	24%	2%
	F1 score	9%	14%	17%	5%	36%	30%	4%
Overall A/H5N1 cases (H+V)	Detected	9	11	11	11	9	11	21
	Not detected	20	18	18	18	20	18	8
	Inadequately detected (e)	94	84	115	336	28	69	767
	Detection rate	31%	38%	38%	38%	31%	38%	72%
	EPPV	9%	12%	9%	3%	24%	14%	3%
	F1 score	14%	18%	14%	6%	27%	20%	5%

*Auto: Automatically emailed; Prov: Provided by system.*

*(a) Virtual combined system pooling the 6 systems i.e. event detected by any of the system was considered as detected by the combined system, (d) differs from (b) + (d) because it includes events that could not be categorized in human cases or epizootics.*

For the six systems, the overall detection rate (DR) ranged from 31% to 38%, from 29% to 57% for human cases and from 20% to 40% for epizootics. Differences in DR were observed between human cases and epizootic events (the largest being 57% for human cases versus 20% for epizootics). For the combined system (pooled from six systems), the DR increased to 72% overall, to 93% for human cases and to 53% for epizootics. Overall EPPV ranged from 3% to 24% and the F1-score ranged from 6% to 27%. The overall EPPV and F1-scores of the combined system were 3% and 5%, respectively.

#### Sensitivity and Timeliness

**Figure 2:**
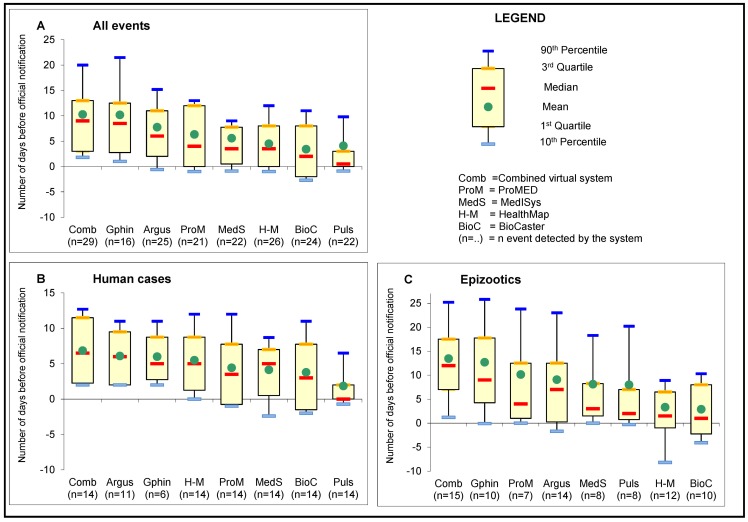
Timeliness of the systems for A/H5N1 cases (total, human, epizootic) reported in March 2010.

Two events (7%) were not detected by the systems before official notification, 6 (21%) events were detected by only one system and only 2 (7%) were detected by the seven systems (Table 4). Sensitivity ranged from 38% to 72% for overall A/H5N1 events, from 29% to 79% for human cases and from 33% to 67% for epizootics. For five systems the sensitivities were higher for human cases than for epizootics. When considering the virtual combined system (seven systems) overall sensitivity increased to 93%, 100% for human cases and 87% for epizootics (Table 5). Timeliness for human cases detected by the systems varied from 1.9 days (confidence interval 95%: −0.4; 4.1) to 6.1 days (3.1; 9.1) before the reporting by WHO. For epizootics the mean timeliness varied from 2.9 days (−3.9; 9.7) to 12.7 days (3.4; 22.0) before OIE reporting. Overall timeliness ranged from 2.2 days (0.5; 3.8) to 7.8 days (4.0; 11.5) before WHO/OIE reporting. Differences observed among systems were not significant (F-statistic calculated for analysis of variance (ANOVA)  = 0.553). For the combined system (pooled from seven systems), events were detected on average 10.2 days (6.7; 13.8) before their reporting by WHO/OIE, while timeliness for human cases was 6.9 days (4.2; 9.5) and 13.5 days (7.1; 19.9) for epizootics (Figure 2).

**Table 4:** Number of gold standard events detected by the systems.

	Not detected	Detected by						
		1 system	2 systems	3 systems	4 systems	5 systems	6 systems	7 systems
n	2	6	3	1	2	8	5	2
%	7%	21%	10%	3%	7%	28%	17%	7%

**Table 5:** Sensitivity of the systems for A/H5N1 cases (overall, human, epizootic) notified by WHO and OIE in March 2010.

A/H5N1 information (raw signals)		Argus	BioCaster	GPHIN	HealthMap	MedISys	ProMED	Puls	Combined System (a)
A/H5N1 human cases	TP	11	9	4	11	10	9	5	14
	FN	3	5	10	3	4	5	9	0
	Se	79%	64%	29%	79%	71%	64%	36%	100%
A/H5N1 epizootics	TP	10	6	8	7	6	5	6	13
	FN	5	9	7	8	9	10	9	2
	Se	67%	40%	53%	47%	40%	33%	40%	87%
Overall A/H5N1 events	TP	21	15	12	18	16	14	11	27
	FN	8	14	17	11	13	15	18	2
	Se	72%	52%	41%	62%	55%	48%	38%	93%

*(a) Virtual combined system pooling the 7 systems i.e. event detected by any of the system was considered as detected by the combined system.*

*TP  =  True positive; FN =  False Negative, Se =  Sensibility.*

## Discussion

The results highlight how combining the expertise of multiple epidemic intelligence systems could substantially increase sensitivity and timeliness. When the seven systems were pooled, the sensitivity of the combined system increased to 93% and events were detected on average 10.2 days earlier (−1; 44 days), a period of time that would indeed be crucial for implementation of control measures in the case of a potential threat. The results also point out the many challenges faced by the system, including the lack of specificity of raw information, and the advances that need to be achieved in this domain.

The qualities most frequently quoted by users in the qualitative analysis were simplicity, usefulness and timeliness while flexibility, representativeness and completeness received much lower scores. The findings were corroborated by practices as all users routinely utilized four to seven different systems. Not all interviewees were familiar with all systems and their routine utilization varied. Although the global approach was similar, each institution has set objectives and procedures that best suit their specifics needs. These differences in both system design and user practice may have influenced the perceptions. Intensively-used systems were more susceptible to being assessed on users' experience and according to their ability to meet an institution's goal (as opposed to their intrinsic performance), while more theoretical opinions might have been applied for seldom used systems. The number of interviewees may appear as a limitation. However, the number of institutions performing structured epidemic intelligence was very limited and the people interviewed were key experts in their domain. It is therefore unlikely that their views substantially differed from those of the team and the institution they represented. We believe that it is unlikely that adding a few additional people or institutions would have resulted in significantly different results.

It must be stressed that system's designs and functionalities are very different [21]. The quantitative analysis was not designed to compare systems' performances and therefore comparison would be misleading. Rather, the objective was to detect and document diversity and potential complementarities from the end-user perspective. Interpretations of these results should therefore avoid pairwise system comparisons.

The goal of computing the detection rate was to assess the capability of a system and the aptitude for a user to detect relevant information from systems in a situation resembling real life conditions. DR provides an estimate of the events adequately tagged A/H5N1 during this period and detected as such by the analyst. However, in order to have the same denominator (also used for the EPPV estimation), events that could have been detected before March 2010 were not included. The calculated DRs are likely to be underestimated and hence should not be regarded as a proxy for sensitivity. The overall DRs were very similar (from 31% to 38%). These low scores could be attributed to the non-inclusion of signals detected before March 1, 2010, but also to the difficulty for an end-user to prospectively detect relevant information in a large volume of noise.

The low measured EPPV and F1-scores illustrate the varying ability of systems to adequately detect, efficiently sort-out, and make accessible only the pieces of information relevant for epidemic intelligence purposes while reducing the background noise. The F1-score [16], by weighing them equally, can provide good balance between EPPV and DR. System developers can increase the F1 score by improving signal detection (e.g., expanding geographical coverage, languages, sources, etc.) and or by reducing background noise (e.g., algorithms for de-duplication). In this study, the F1 score was strongly impacted by the high numbers of XDET and the EPPV, which can lower the sensitivity performance (DR). In a period of one month and considering only one clearly identified topic, A/H5N1, 1,154 documents were detected by the seven systems (on average 37 per day), hence providing an indication of the volume of information to be reviewed routinely when extended to an all hazard approach (i.e., covering all potential health threats). The EPPV of the virtual combined system was very low, however a genuine operational combined system would include functionalities (e.g., de-duplication) that would substantially reduce the redundant information, hence increasing performance.

No single system was able to detect all events included in the gold standard before their public reporting by WHO or OIE. Sensitivity varied from 38 to 72%. An average difference of 23% was observed between the sensitivities calculated for human cases and epizootics (Table 4) but no explanation was found for such a large difference within and across systems. These findings, however, underline the difference in conceptual design and the associated performance, but also the difficulties met in developing an efficient algorithm covering the different facets of a single disease.

No significant difference (ANOVA  = 0.553) was observed between system timeliness. The difference in the number of detected events could have contributed to the observed variation. Systems operated in different time zones and normalizing time proved difficult (because time of posting was not retrievable for all systems). Although for the systems for which information was available, no difference was observed, an effect of the time of posting could not be formally ruled out. Nevertheless, our findings are consistent with other studies: HealthMap detected events around 12 days before WHO publication and ProMED-mail between 2 days and 2 weeks earlier than OIE when events were detected by both sources [22], Argus detected confirmed cases of pandemic (A/H1N1) from 1 to 16 days ahead of WHO for 42 countries [40]. No timeliness differences were found between HealthMap, BioCaster and EpiSPIDER [20].

A number of limitations have been identified in this study. The first one concerns the gold standard. The choice of A/H5N1 events was suggested by its public health significance and the existence of an easy to access gold standard. Across affected countries, access to health care, laboratory facilities, surveillance systems, national protocols for biological confirmation (for both human and animal diseases), control strategies vary greatly and not all events will have samples submitted for biological confirmation. Finally, reporting by both the WHO and OIE is subjected to an official notification by a national authority, a process that can take time and that is not always performed. The limits of using WHO and OIE as a gold standard have already been pointed out by previous studies [22]–[25], though very few surveys proposed alternatives [26], [27]. It is likely that only a portion of genuine A/H5N1 occurrences was effectively reported to WHO or OIE but the magnitude of this bias cannot be estimated. Measured values (DR, EPPV and F1-score) could have been underestimated. Nevertheless, reports classified as XDET were often duplicates (redundant information) or misclassified reports (not related to A/H5N1 cases) as opposed to non-verified events. The impact on the EPPV and F1-score is likely to have been limited while the effect of this potential bias might be more important for DR and Se. It cannot be assumed that the weight of the bias was evenly distributed and that the performances of individual systems were likely to have been affected in different ways.

Despite the heterogeneity of designs [21] the same methodology had to be used for all systems. This uniform approach allowed for the provision of a global overview, but did not reflect adequately the large variability of systems' functionalities and genuine performances. The systems are in constant evolution (internal methodology, algorithms, etc.), but for a short study period such changes are likely to be minor and not impact the results. This study was implemented in the scope of EAR and the results had to be delivered within a fixed time frame. The assessment was thus intentionally performed over a short period and was focused on only one topic (A/H5N1) in order to keep the number of signals relatively small. This evaluation could not integrate all potentially important elements, such as languages, geographical distribution, type of sources, interconnections among systems, and others. Excluding such parameters may limit the results generalizability, but despite these limitations and potential biases, the results provided a global perspective and a characterization of the complexity of epidemic intelligence under “real life” conditions. In the scope of EAR, the study results helped to inform future research strategies, i.e., identifying each system's strengths and defining mechanisms that will allow more efficient synergies and cross-fertilization of knowledge and information as opposed to attempting to strengthen “the best of the systems” or to create a “new system”.

## Conclusions

This study emphasized the added value, and synergistic qualities: between systems, among users and between systems and users. The complexity and the diversity of the epidemic intelligence approaches and the vast expertise developed by the systems are much broader than what could be described in this article [9]. Despite the systems' success, both systems and institutions face major challenges [28] such as the rapidly escalating volume of Internet information, the changing type of communication and information dissemination (i.e., social networks and brief, instantaneous communications) and the management of large volumes of data. Levels of duplicative information and noise are very high and international collaboration is still limited. No super-system exists to pool expert systems' expertise and more initiatives must be developed in this direction. More research needs to be carried out, including longer study periods, different types of health events and more robust gold standards. Also additional users and other systems' perspectives should be considered. Overall, this relatively easy to implement study constitutes a first step that will hopefully pave the way for continued exploration in this challenging, but essential component of the global and nations' health security processes and initiatives.
